# *Lignosus rhinocerotis* (Cooke) Ryvarden mimics the neuritogenic activity of nerve growth factor via MEK/ERK1/2 signaling pathway in PC-12 cells

**DOI:** 10.1038/srep16349

**Published:** 2015-11-06

**Authors:** Syntyche Ling-Sing Seow, Lee-Fang Eik, Murali Naidu, Pamela David, Kah-Hui Wong, Vikineswary Sabaratnam

**Affiliations:** 1 Mushroom Research Centre, Faculty of Science, University of Malaya, Kuala Lumpur, Malaysia; 2 Institute of Biological Sciences, Faculty of Science, University of Malaya, Kuala Lumpur, Malaysia; 3 Department of Anatomy, Faculty of Medicine, University of Malaya, Kuala Lumpur, Malaysia

## Abstract

The traditional application of the sclerotium of *Lignosus rhinocerotis* (tiger’s milk mushroom) by the indigenous folks as tonic and remedy to treat a variety of ailments has been documented in Malaysia. Indigenous communities claimed to have consumed the decoction to boost their alertness during hunting. Mental alertness is believed to be related to neuronal health and neuroactivity. In the present study, the cell viability and neuritogenic effects of *L. rhinocerotis* sclerotium hot aqueous and ethanolic extracts, and crude polysaccharides on rat pheochromocytoma (PC-12) cells were studied. Interestingly, the hot aqueous extract exhibited neuritogenic activity comparable to NGF in PC-12 cells. However, the extracts and crude polysaccharides stimulated neuritogenesis without stimulating the production of NGF in PC-12 cells. The involvements of the TrkA receptor and MEK/ERK1/2 pathway in hot aqueous extract-stimulated neuritogenesis were examined by Trk (K252a) and MEK/ERK1/2 (U0126 and PD98059) inhibitors. There was no significant difference in protein expression in NGF- and hot aqueous extract-treated cells for both total and phosphorylated p44/42 MAPK. The neuritogenic activity in PC-12 cells stimulated by hot aqueous and ethanolic extracts, and crude polysaccharides of *L. rhinocerotis* sclerotium mimicking NGF activity via the MEK/ERK1/2 signaling pathway is reported for the first time.

Medicinal mushrooms and their extracts have a long and rich history of use in traditional oriental medicines as mycomedicines^1^. Increasingly, many are being regarded as functional foods and nutraceuticals. The neuroactivities of medicinal mushrooms are under intense study and research. Phan *et al.* (2014)^2^ reviewed a number of studies of medicinal mushrooms, revealing the promises of medicinal mushrooms as useful therapeutic agents in the management and/or treatment of neurodegenerative disorders.

In Malaysia, *Lignosus rhinocerotis* (Cooke) Ryvarden is also known as ‘tiger’s milk mushroom’ in English or ‘cendawan susu rimau’ in Malay. It is considered as a unique “National Treasure” that can only be found in a small geographic region in Southern China, Thailand, Malaysia, Indonesia, Philippines, Papua New Guinea, New Zealand and Australia^3^. In Malaysia, *L. rhinocerotis* is the most popular medicinal mushroom used by the indigenous communities of Peninsular Malaysia^4^. The benefits of its underground tuber or sclerotium (where most of the nutritional and medicinal components are deposited) compared to its basidiocarp are well documented (Table 1). According to the ethnopharmacological reports, the sclerotium is sliced, boiled and drunk as an effective tonic for overall wellness and to treat several ailments including fever, cough, asthma, chronic hepatitis, gastric ulcer, cancer and food poisoning^4^,^5^,^6^,^7^ (Table 1). Indigenous communities also drank this decoction to increase their alertness during hunting (tacit knowledge). Moreover, Tan *et al.* (2012)^3^ documented that the consumption of tiger’s milk mushroom improved stamina and alertness in healthy people. Increment of the mental alertness is believed to be related to the neuroactivity and neuronal communication network in brain. Based on the traditional practice as the basic for the scientific study, we explored *L. rhinocerotis*, the Malaysia’s treasure mushroom, notably for the neuritogenic activity *in vitro*.

Neuritogenesis is a dynamic phenomenon associated with neuronal differentiation, where the neurons generate and extend their neurites to form a functional network^8^. Neuritogenic activity is important for the maintenance and regeneration of the neuronal communications network and has become one of the focuses of study in the search for preventive and therapeutic agents for neurodegenerative disorders. The neurotrophic factors (neurotrophins), exemplified by nerve growth factor (NGF), play distinctive roles in promoting neuronal survival^9^, proliferation, development, neuritogenesis and maintaining the neurons functions^10^. Nerve growth factor was identified as a potential therapeutic agent for the treatment of neurodegenerative disorders^11^. A decrease in NGF in the brain is believed to be the main cause of neuronal dysfunction and neurodegenerative disorders, remarkably Alzheimer’s disease^12^. Further, Capsoni *et al.* (2000)^13^ presented the evidence that a decrease in NGF in mice’s brain led to neurodegeneration and Alzheimer’s-like symptoms. Learning ability and memory were improved in aged anti-nerve growth factor transgenic mice after administration of NGF^13^. However, the neuroactivity of NGF is restricted due to its large molecular polypeptide structure. It is unstable and unable to cross the blood-brain barrier^14^. Smaller molecules that mimic and/or enhance the NGF activity have become the core focus in the search for preventive and therapeutic agents for neurodegenerative disorders^15^. Among the natural sources explored for NGF mimics, medicinal mushrooms have shown huge potential^2^.

Nerve growth factor was found to activate the mitogen-activated protein kinase (MAPK) signaling pathway that mediates the phosphorylation including the mitogen-activated protein kinase kinase/extracellular signal-regulated kinase (MEK/ERK) pathway^16^. The MAPK/ERK pathway is a chain of proteins in the cell that participate by Ras, Raf, MEK1/2 and ERK1/2 proteins. The MAPK/ERK cascade is a signal transduction pathway that involves a large variety of processes such as differentiation, proliferation, apoptosis, cell cycle progression, cell migration, and metabolism^17^. The MEK/ERK1/2 signalling cascade is indeed crucial in both *in vitro*^18^ and *in vivo* for neuronal cell survival, axonal regeneration^19^ and neuritogenesis in extract-treated mouse dorsal root ganglia neurons^20^.

The MEK/ERK1/2 signaling pathway is believed to be the major cascade for NGF to stimulate neuritogenesis in PC-12 cells^18^. The PC-12 cell line is widely used as an *in vitro* model system to investigate the neuritogenic activity of NGF and NGF mimics, and NGF responsive signaling pathways^15^,^18^,^21^,^22^. Nerve growth factor stimulates differentiation of PC-12 cells into a sympathetic neuronal-like phenotype and extend axon-like outgrowth^23^. PC-12 cells treated with NGF have been found to stop proliferating and differentiate into neuronal-like cells with neurite outgrowth^23^. *In vitro* neuritogenesis stimulated by hot aqueous extract of *Ganoderma* sp.^24^, *Pleurotus giganteus* (Berk.) Karunarathna & K. D. Hyde^25^ and *Ganoderma neo-japonicum* Imazeki^26^ in PC-12 cells were mediated via MEK/ERK1/2 signaling pathway.

A number of studies revealed that the sclerotium of *Polyporus rhinocerus* (synonym of *L. rhinocerotis*) demonstrated anti-tumour^27^,^28^,^29^, immunomodulating^30^,^31^,^32^, and antioxidant^33^ activities in *in vitro* (Table 1). However, very limited information is available on the neuronal activity of *L. rhinocerotis*. Our recent findings revealed that the hot aqueous extract of *L. rhinocerotis* sclerotium stimulated neuritogenesis in PC-12 cells^34^. To further investigate the neuritogenic activity of the *L. rhinocerotis* sclerotium, in the present study, we aimed to (i) compare the cell viability and neuritogenic effects of PC-12 cells of three different preparations, including hot aqueous and ethanolic extracts, and crude polysaccharides of *L. rhinocerotis* sclerotium, (ii) investigate whether the extracts and crude polysaccharides stimulate the production of NGF, and (iii) investigate the involvement of NGF responsive signaling pathway (MEK/ERK1/2) in *L. rhinocerotis* sclerotium-stimulated neuritogenesis in PC-12 cells.

## Results and Discussions

### The effects of hot aqueous and ethanolic extracts, and crude polysaccharides on viability of PC-12 cells

**Figure 1:**
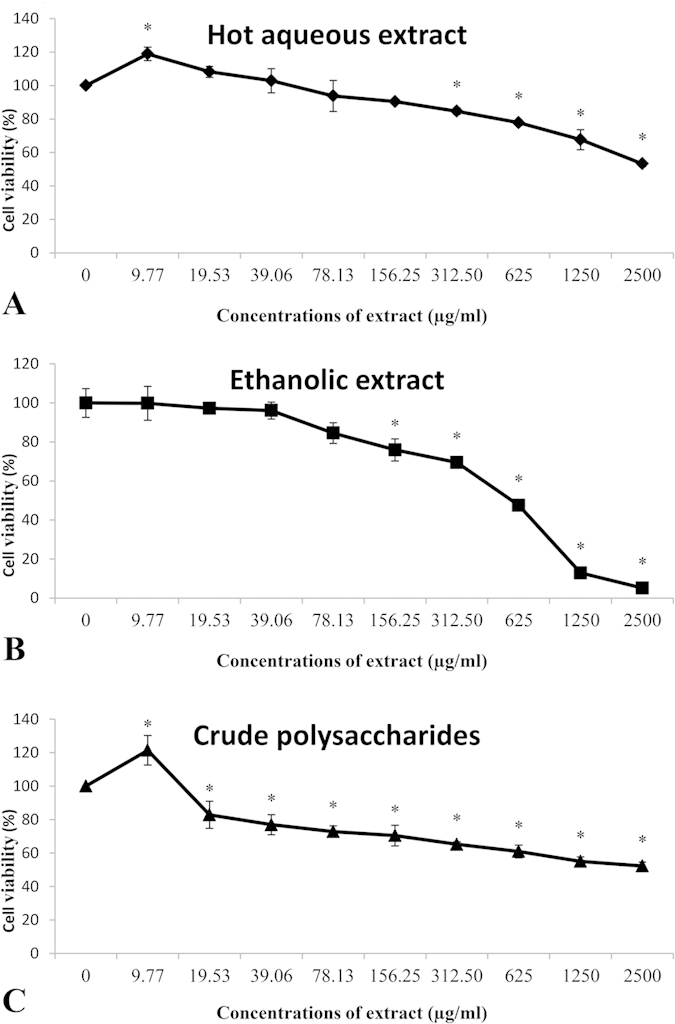
The effects of hot aqueous and ethanolic extracts, and crude polysaccharides of *L. rhinocerotis* sclerotium on viability of PC-12 cells. Cells were incubated with extracts or crude polysaccharides at concentrations from 0 to 2500 μg/ml for 48 h. (**A**) hot aqueous extract, (**B**) ethanolic extract, and (**C**) crude polysaccharides. The mean absorbance obtained using complete F-12 K medium with cells only (negative control) was designated 100% of cell viability. Results are shown as means ± standard deviation (n = 3). *p < 0.05 compared to the respective negative control 100%.

The viability of cells in complete F-12 K medium was considered as 100%. The survival and proliferation of treated cells decreased in a concentration-dependent manner for hot aqueous extract (Fig. 1A), ethanolic extract (Fig. 1B), and crude polysaccharides (Fig. 1C). Hot aqueous extract and crude polysaccharides increased the cells proliferation significantly (p < 0.05) at low concentration, 9.77 μg/ml, compared to the negative control (cells in complete F-12 K medium only). The percentage of viable cells decreased gradually as the concentration of the extracts and crude polysaccharides were increased. The percentage of viable cells in hot aqueous and ethanolic extracts-treated cells were reduced significantly (p < 0.05) starting at 156.25 μg/ml compared to negative control, while the viability of crude polysaccharides-treated cells was reduced significantly (p < 0.05) starting at a low concentration, 19.53 μg/ml, compared to negative control. The required concentrations to inhibit the cell growth by 50% (IC_50_) for hot aqueous, ethanolic extracts, and crude polysaccharides were 3223.98 μg/ml, 372.30 μg/ml and 2718.72 μg/ml, respectively. The cytoxicity of the hot aqueous extract was significantly (p < 0.05) lower in the *in vitro* PC-12 cell model when compared to the crude polysaccharides and ethanolic extract. In a study done by Lee *et al.* (2011, 2013)^35^,^36^, it was reported that in the *in vivo* model, no treatment-related chronic toxicity was detected in Sprague Dawley rats after a long term (180 days) oral administration of the *L. rhinocerotis* sclerotium freeze-dried powder (cultivar TM02) at daily dosage up to 1,000 mg/kg. Further, blood biochemical parameters related to toxicity were reported as normal. In that study, however, Lee *et al.* (2011, 2013) did not estimate the levels of the mushroom in the blood.

### The neuritogenic effects of hot aqueous and ethanolic extracts, and crude polysaccharides in PC-12 cells

**Figure 2:**
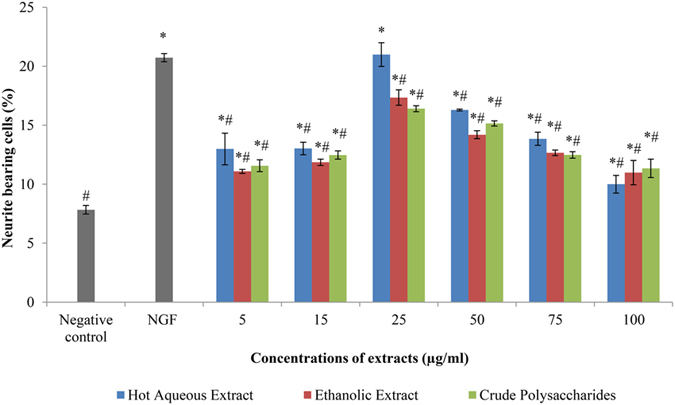
The neuritogenic effects of hot aqueous and ethanolic extracts, and crude polysaccharides in PC-12 cells. Cells were incubated with NGF (50 ng/ml), extracts or crude polysaccharides (5 to 100 μg/ml) for 48 h. Cells in complete F-12 K medium served as a negative control. Cells treated with 50 ng/ml of NGF served as a positive control. Data were expressed as means ± standard deviation (n = 3). *p < 0.05 compared to the negative control. #p < 0.05 compared to the positive control (50 ng/ml of NGF).

Neurite extension of PC-12 cells was regarded as an index of neuritogenesis. All the tested concentrations of hot aqueous and ethanolic extracts, and crude polysaccharides stimulated neuritogenesis in PC-12 cells after 48 h of incubation (Fig. 2). The percentage of neurite-bearing cells in all the tested concentrations of extracts and crude polysaccharides treated cells were increased significantly (p < 0.05) compared to the negative control (cells in complete F-12 K medium only). The percentage of neurite-bearing cells was concentration-dependent (from 5 μg/ml to 100 μg/ml) of extracts and crude polysaccharides tested. There was an initial increase of the percentage of neurite bearing cells on increasing concentrations of extracts and crude polysaccharides, followed by a decrease at concentrations above 25 μg/ml. All the extracts and crude polysaccharides stimulated maximal neuritogenesis in PC-12 cells at 25 μg/ml. The hot aqueous extract stimulated highest percentage of neurite-bearing cells (20.99 ± 1.01%), followed by the ethanolic extract (17.35 ± 0.66%) and crude polysaccharides (16.40 ± 0.26%), at 25 μg/ml. The IC_50_ value of the cytotoxic activity of the hot aqueous extract, ethanolic extract and crude polysaccharides and was approximately 129-, 15- and 109- fold higher than their optimum concentration that stimulated neuritogenesis, 25 μg/ml. Our previous study^34^ reported that 50 ng/ml of NGF was the optimum concentration for neuritogenesis in PC-12 cells. In the present study, cells treated with 50 ng/ml of NGF served as a positive control. Interestingly, there was no significant difference (p > 0.05) in the percentage of neurite-bearing cells between 50 ng/ml of NGF- and 25 μg/ml of hot aqueous extract-stimulated neuritogenesis. This is in agreement with the previous studies, reported that 20 μg/ml of hot aqueous extract of *L. rhinocerotis* sclerotium^34^ and *L. rhinocerotis* mycelium^37^ stimulated neuritogenesis in PC-12 cells that comparable to NGF.

The hot aqueous extraction is the most commonly used method by the indigenous communities and traditional Chinese medicine (TCM) physicians to prepare decoctions, tonics or essences from medicinal mushrooms. In general, mushroom’s polysaccharides are targeted as the active components in the hot aqueous extract^38^. Mushroom’s polysaccharides include α-glucans and β-glucans, that known as effective anti-tumor and immunomodulatory agents^39^. Besides carbohydrates and proteins, Lau *et al.* (2013)^29^ reported that the hot aqueous extract of *L. rhinocerotis* sclerotium contained a higher percentage of phenolics than the cold aqueous extract. Lau *et al.* (2013)^29^ further suggested that secondary metabolites such as triterpenes and alkaloids may be present in *L. rhinocerotis* sclerotium extracts. Secondary metabolites isolated from different mushrooms that were reported to promote neuritogenesis *in vitro* and/or *in vivo*, include hericenones^40^, erinacines^41^, scabronions^42^, termitomycesphins^43^ and cyrneines^44^. These neuroactive compounds may be targeted as preventive and therapeutic agents for neurodegenerative disorders. In the present study, the hot aqueous extract of *L. rhinocerotis* sclerotium showed potent neuritogenic activity compared to the ethanolic extract and crude polysaccharides. These findings suggested that the hot aqueous extract containing chemical constituents that are neuroactive.

### The morphology of PC-12 cells stained with anti-neurofilament-200 antibody

**Figure 3:**
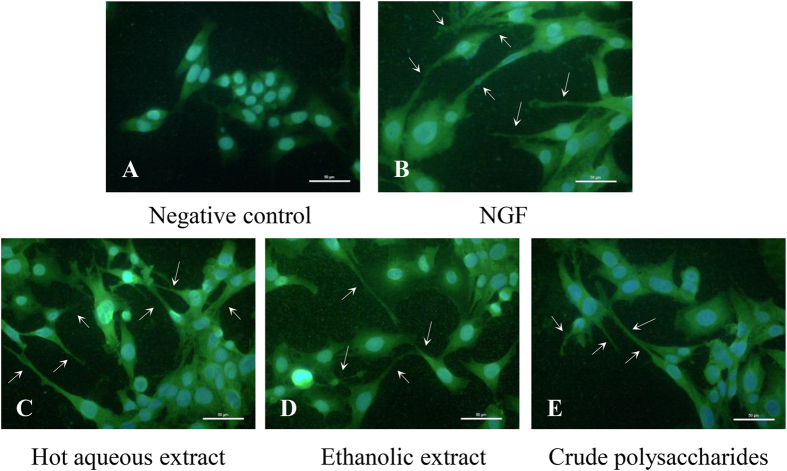
Morphology of PC-12 cells stained with anti-NF-200 antibody. Cells were incubated with or without NGF (50 ng/ml), hot aqueous extract (25 μg/ml), ethanolic extract (25 μg/ml) or crude polysaccharides (25 μg/ml) for 48 h. Cells in complete F-12 K medium served as a negative control. Cells treated with 50 ng/ml of NGF served as a positive control. Nuclei stained blue and neurofilaments stained green. Scale bars represent 50 μM. Arrows indicate neurite outgrowth.

Neurofilaments are neuron specific intermediate filament proteins (8–10 nm) that are located in axons, found specifically in most mature neurons^45^. Neurofilaments are composed predominantly of distinct subunits, namely neurofilament light (NF-L), medium (NF-M) and heavy (NF-H)^46^. The anti-NF-200 antibody recognizes both phosphorylated and non-phosphorylated forms of heavy neurofilament subunit NF-H at 180–220 kDa. Immunostaining of neurofilaments confirmed the neuritogenesis was stimulated by NGF, hot aqueous, ethanolic and crude polysaccharides extracts (Fig. 3). PC-12 cells nuclei were stained blue by DAPI and neurofilaments were stained green by anti-NF-200 antibody labelled with FITC. Cells were elongated and exhibited significant neurite extensions in NGF-, hot aqueous extract-, ethanolic extract- and crude polysaccharides-stimulated neuritogenesis.

### The concentration of extracellular NGF of hot aqueous and ethanolic extracts-, and crude polysaccharides-treated cells

**Figure 4:**
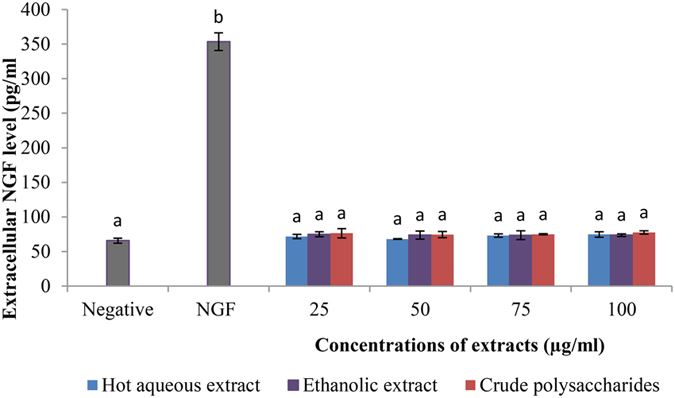
Extracellular NGF concentration in supernatants of NGF-, extracts- or crude polysaccharides-treated PC-12 cells. Cells were incubated with or without NGF (50 ng/ml), extracts or crude polysaccharides (25 to 100 μg/ml) for 48 h. Cells in complete F-12 K medium served as a negative control. Cells treated with 50 ng/ml of NGF served as a positive control. Data were expressed as means ± standard deviation (n = 3). Means with different alphabets show significant difference (p < 0.05).

The increase in extracellular NGF in cell supernatant showed the ability of tested compounds to induce NGF production by PC-12 cells^47^. The concentration of extracellular NGF of PC-12 cells without treatment (negative control) was detected at 65.64 pg/ml (Fig. 4). The concentration of extracellular NGF in 50 ng/ml of NGF-treated cells (positive control) was 353.42 pg/ml, which was approximately five fold increase compared to the negative control. However, there was no significant difference (p > 0.05) in the concentration of extracellular NGF between all the tested concentrations of the extracts and crude polysaccharides, and the negative control.

The present findings showed that the concentration of extracellular NGF in 50 ng/ml of NGF-treated cells increased by approximately 81% compared to the non-treated cells. Based on the amount of extracellular NGF measured and percentage of neurite bearing cells observed in positive control (NGF-treated cells), neuritogenesis in PC-12 cells may be NGF-dependent. Number of studies documented the potential of extracts and compounds of edible and medicinal mushrooms to stimulate the biosynthesis and secretion of NGF *in vitro*^40^,^41^,^42^,^44^,^47^,^48^. According to Lai *et al.* (2013)^48^, hot aqueous extract of *Hericium erinaceus* (Bull.: Fr.) Pers. (lion’s mane mushroom) stimulated the production of NGF in NG108–15 cells, a hybrid neuronal cell line derived from mouse neuroblastoma and rat glioma. The concentration of the extracellular NGF in NG108–15 cells treated with 50 μg/ml of hot aqueous extract of *H. erinaceus* was 21.4% higher, compared to the cells treated with 20 ng/ml of NGF^48^. Compounds from *H. erinaceus*, including hericenone C-E^40^, erinacine A-C^41^ stimulated biosynthesis of NGF and exhibited neuritogenesis in astroglial cells. In a recent study by Phan *et al.* (2014)^47^ reported that hericenones E potentiated NGF-induced neuritogenesis in PC-12 cells by stimulating the production of NGF that was almost two times higher than that of positive control (50 ng/ml of NGF). However, in the present study, *L. rhinocerotis* sclerotium extracts and crude polysaccharides stimulated the neuritogenic activity without stimulating the production of NGF in PC-12 cells. These findings showed that the extracts might contain NGF-like compound(s) (NGF mimics or substitute for NGF) that mimic the neuritogenic activity of the NGF.

### The involvement of NGF responsive signaling pathway in hot aqueous extract-stimulated neuritogenesis

**Figure 5:**
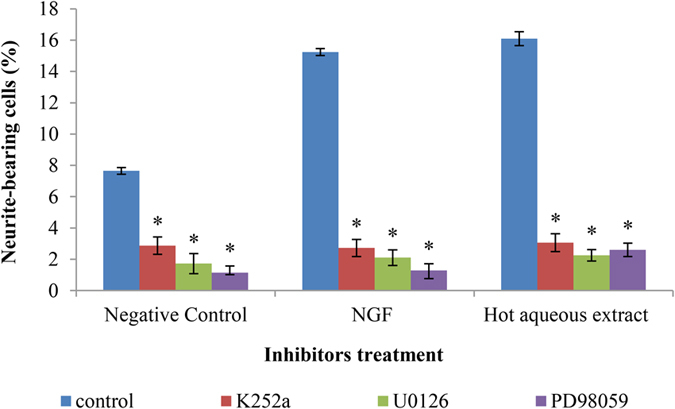
The effects of the specific inhibitors of TrkA and MEK/ERK1/2 on NGF-, or hot aqueous extract-stimulated neuritogenesis. Cells were pre-treated with K252a, U0126, PD98059 for one hour before the treatment with NGF (50 ng/ml) or hot aqueous extract (25 μg/ml). Cells in complete F-12 K medium served as a negative control. Cells treated with 50 ng/ml of NGF served as a positive control. A control (without inhibitor) was used in each treatment group. Data were expressed as means ± standard deviation (n = 3). *p < 0.05 compared to the respective controls.

The NGF responsive pathway, TrkA-MEK1/2-ERK1/2 was selected as the targeted cascade for neuritogenic activity in PC-12 cells. The Trk and MEK/ERK1/2 inhibitors, namely K252a, U0126 and PD98059 significantly (p < 0.05) blocked the NGF- and hot aqueous extract-stimulated neuritogenesis (Fig. 5). The K252a, U0126 and PD98059 decreased the percentage of neurite-bearing cells by approximately 82.13%, 86.15% and 91.56% in NGF-treated cells, and 80.97%, 86.68% and 84.59% in hot aqueous extract-treated cells, respectively. The significant (p < 0.05) reduction of neurite stimulation activity was also observed in the negative control with the addition of the inhibitors.

TrkA is a cell surface transmembrane receptor tyrosine kinase for NGF and activated TrkA is critical for activation of the Ras/MAPK signaling pathway^49^,^50^. Nerve growth factor binds to its high affinity receptor, TrkA to initiates the NGF responsive pathways, such as the MEK/ERK1/2 signaling pathway, to kick start neuritogenesis. Once the TrkA was phosphorylated, it became a scaffolding structure and recruits proteins that ultimately propagate the MEK/ERK signaling pathway^51^. Specific inhibitors of protein kinase served as powerful tool to study the kinase activities in selected signaling pathway^2^. K252a acts as a specific and potent inhibitor of Trk receptor, inhibits the phosphorylation of NGF-induced TrkA, and selectively blocks the activities of NGF in PC-12 cells^52^. In the present study, the neuritogenic activity of hot aqueous extract was blocked 80.97% by K252a, parallel to the inhibition effect of K252a (82.13%) towards NGF-treated cells. These findings showed that the hot aqueous extract-stimulated neuritogenesis was TrkA-dependant in PC-12 cells.

Both U0126 and PD98059 are selective and potent inhibitors of MEK 1 and MEK 2^53^. Priming PC-12 cells with U0126 and PD98059 will inhibit the phosphorylation and activation of MEK/ERK1/2, and eventually diminish cell differentiation and neuritogenesis^54^. Nishina *et al.* (2006)^55^ demonstrated that the activation of MAPK by lysophosphatidylethanolamine, a neuroactive compound extracted from *Grifola frondosa* (maitake mushroom) was suppressed by U0126, but not by K252a. The study suggested that the MEK/ERK1/2 signaling pathway was involved in lysophosphatidylethanolamine-induced neuritogenesis in PC12 cells, but was not through the activation of TrkA receptor^55^. Phan *et al.* (2014)^47^ reported that the neuritogenic activity potentiated by hericenone E was found to be partially blocked (46%) by K252a and almost completely blocked by U0126 and PD98059, suggested that hericenone E potentiated NGF-stimulated neuritogenesis in PC12 cells was partially mediated by TrkA and MEK/ERK1/2 dependent. In the present study, all three inhibitors (K252a, U0126 and PD98059) successfully attenuated the NGF- and hot aqueous extract-stimulated neuritogenesis in PC-12 cells, showed that the hot aqueous extract of *L. rhinocerotis* sclerotium mimicked the NGF neuritogenic activity by binding to the TrkA receptor and activated the MEK/ERK1/2 signaling pathway in PC-12 cells.

### The protein expression of total p44/42 MAPK (ERK1/2) and phosphorylated p44/42 MAPK (Thr202/Tyr204) in hot aqueous extract-treated cells

**Figure 6:**
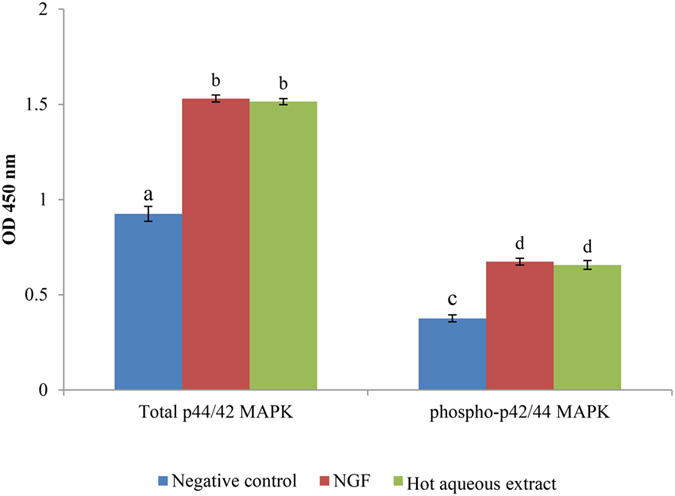
Protein expression of total p44/42 MAPK (Erk1/2) and phosphorylated p44/42 MAPK (Thr202/Tyr204) activation in PC-12 after 48 h of incubation with NGF or hot aqueous extract. Cells were incubated with or without NGF (50 ng/ml) or hot aqueous extract (25 μg/ml) for 48 h. Cells in complete F-12 K medium served as a negative control. Cells treated with 50 ng/ml of NGF served as a positive control. Total p44/42 and phosphorylated p44/42 protein level were measured by determination of the optical intensity (OD). Data were expressed as means ± standard deviation (n = 3). Different alphabets indicate significant difference (p < 0.05).

Endogenous level of total p44/42 MAPK (ERK1/2) and phosphorylated p44/42 MAPK (Thr202/Tyr204) were quantified by ELISA. The magnitude of absorbance for the developed colour is proportional to the quantity of MAPK protein expressed by PC-12 cells. The expression of both total and phosphorylated p44/42 MAPK protein in PC-12 cells treated with NGF and hot aqueous extract were significantly (p < 0.05) higher compared to the negative control (Fig. 6). Both total and phosphorylated p44/42 protein concentrations in NGF-treated cells were higher compared to the hot aqueous extract-treated cells and negative control (p < 0.05). However, there was no significant difference (p > 0.05) in protein expression between NGF- and hot aqueous extract-treated PC-12 cells for both total and phosphorylated p44/42, pERK1 and pERK2.

Activation of NGF responsive pathway by mushroom extract is crucial in the preliminary search of neuroactive compound(s) that may mimic the neuritogenic activity of NGF. Activation of MEK/ERK1/2 provided the biochemical evidence for neuritogenesis and the presence of neurite-stimulating agent(s) in the extract. Cheung *et al.* (2000)^24^ reported that *G. lucidum* aqueous extract promoted neuritogenic and neuroprotective activity via Ras/ERK pathway in PC-12 cells, by demonstrating the phosphorylation of ERK1 and ERK2. In the present study, the endogenous level of total and phosphorylated p44 (ERK1) and p42 (ERK2) proteins were elevated, suggesting that the phosphorylation and activation of ERK1/2 were involved in the stimulation of neuritogenesis in PC-12 cells by the hot aqueous extract of *L. rhinocerotis* sclerotium.

### Immunofluorescence study demonstrated the protein expression of phosphorylated p44/42 MAPK (Thr202/Tyr204) in hot aqueous extract-treated cells

**Figure 7:**
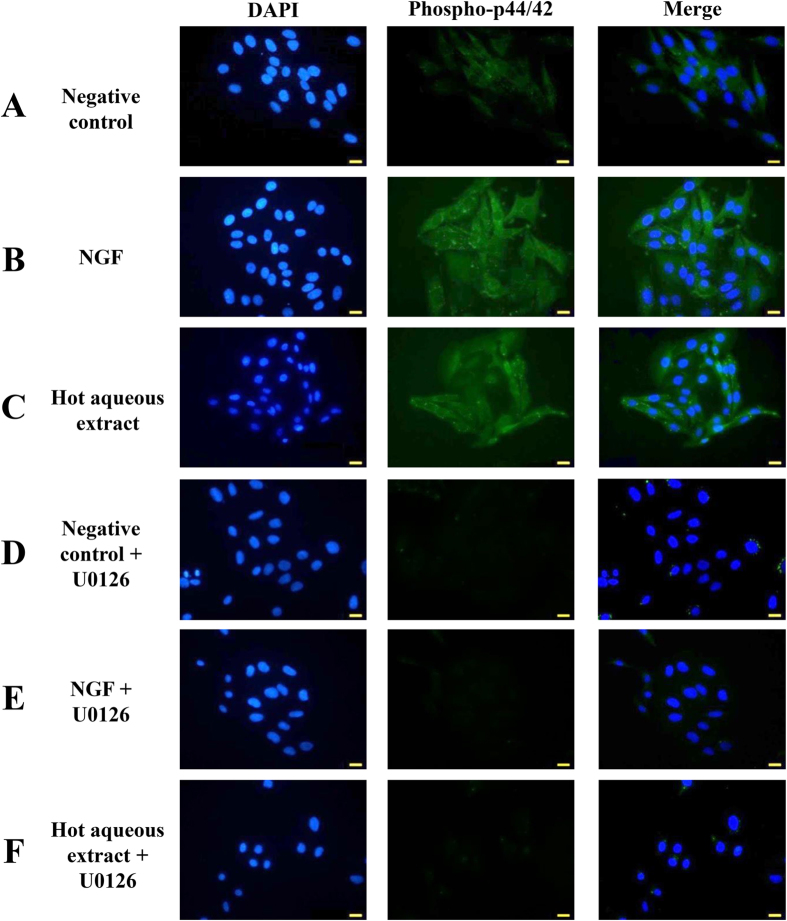
Immunocytochemistry study of phosphorylated p44/42 MAPK (Thr202/Tyr204) in NGF- or hot aqueous extract-treated PC-12 cells after 48 h of incubation. (**A**) Negative control, cells in complete F-12 K medium only. (**B**) Positive control, cells treated with 50 ng/ml of NGF. (**C**) Cells treated with 25 μg/ml of hot aqueous extract. (**D**–**F**) Cells were pre-treated with 10 μM U0126 prior to the treatment with NGF or hot aqueous extract. Phosphorylated p44/42 protein stained cell body in green. DAPI stained nuclei in blue. Scale bar = 50 μm.

Immunofluorescence staining was served as a visualize support to the protein expression of phosphorylated p44/42 MAPK (Thr202/Tyr204) (Fig. 7). The intensity of the immunofluorescence staining demonstrated the protein expression level of phosphorylated p44/42 protein in PC-12 cells. The intensity of immunofluorescence staining of phosphorylated p44/42 protein was higher in NGF- (Fig. 7B) and hot aqueous extract- (Fig. 7C) treated cells than the negative control (Fig. 7A). An MEK/ERK1/2 inhibitor, U0126 was used as a control to ensure the involvement of MEK/ERK1/2 signaling pathway in neuritogenesis. Pre-treatment with U0126 in PC-12 cells showed lower signal intensity (Fig. 7D–F). Results showed that neuritogenesis in PC-12 cells was dependent on the activation of MEK/ERK1/2 signaling pathway.

## Conclusions

The hot aqueous and ethanolic extracts, and crude polysaccharides of *L. rhinocerotis* sclerotium stimulated neuritogenesis in PC-12 cells. All the concentrations of the extracts and crude polysaccharides tested for neuritogenic activity were not cytotoxic to PC-12 cells. The hot aqueous extract (25 μg/ml) stimulated neuritogenic activity that was comparable to NGF (50 ng/ml). The extracts and crude polysaccharides stimulated neuritogenic activity but did not stimulate the production of NGF in PC12 cells. The neuritogenic activity of NGF- and hot aqueous extract may be mediated through the phosphorylation of TrkA receptor and ERK1/2 signaling pathway in PC-12 cells. *Lignosus rhinocerotis* sclerotium may contain neuroactive compound(s) that mimic the neuritogenic activity of NGF, and induce neuritogenesis in PC-12 cells via the NGF responsive pathway, TrkA-MEK1/2-ERK1/2 signaling pathway.

## Methods

### Preparation of hot aqueous and ethanolic extracts, and crude polysaccharides of *L. rhinocerotis* sclerotium

*Lignosus rhinocerotis* sclerotium light brown and dry fluffy freeze-dried powder (LiGNO™ cultivar TM02) was purchased from Ligno Biotech Sdn. Bhd., Malaysia^56^. Every batch of cultivar TM02 freeze-dried powder is identified and validated by the internal transcribed spacer regions of ribosomal RNA^57^. The hot aqueous extraction was carried out according to Wong *et al.* (2007)^58^ with modification. Briefly, the freeze-dried powder was soaked in distilled water at a ratio of 1:20 (w/v) and was agitated on a shaker at 150 rpm at room temperature, overnight. Then, the mixture was double boiled in a water bath for 30 min, and cooled to room temperature. The mixture was then centrifuged at 7,800 × g for 15 min, and the supernatant was collected and filtered through Whatman no. 4 filter paper. The resulting hot aqueous extract was freeze-dried and kept at −20 °C prior to use. For the ethanolic extract, the freeze-dried powder was soaked in 80% ethanol (v/v in distilled water) at room temperature for three days and the process was repeated three times. The ethanol was evaporated using a rotary evaporator (Eyela N-1000). The resulting ethanolic extract was kept in −20 °C prior to use. The crude polysaccharides were extracted according to the alkaline extraction method of Ojha *et al.* (2010)^59^. The freeze-dried powder was soaked in 4% (w/v) sodium hydroxide (NaOH) and heated in 80 °C water bath for 45 min, then the mixture was centrifuged at 7,800 × g for 45 min. Supernatant was collected and precipitated with absolute ethanol at a ratio of 1:5 (v/v). The mixture was kept for 12 h at 4 °C. The precipitated polysaccharides were centrifuged at 7,800 × g for 45 min. The residue was dialysed using diethylaminoethyl (DEAE) cellulose bag for 4 h. The crude polysaccharides were freeze-dried and kept at −20 °C prior to use.

### *In vitro* cell culture

The PC-12 cells (American Type Culture Collection, ATCC) were maintained in complete Kaighn’s Modification of Ham’s F-12 (F-12 K) Medium (Sigma) supplemented with 15% of heat-inactivated horse serum and 2.5% of heat-inactivated Fetal bovine serum (PAA Laboratories) at 37 ± 2 °C in a 5% CO_2_-humidified incubator. The cells were passaged every 2 to 3 days upon 80% confluent.

### Assessment of the effects of hot aqueous and ethanolic extracts, and crude polysaccharides on viability of PC-12 cells

Cells were plated at a density of 1 × 10^4^ cells per well in 96-well plates and incubated overnight at 37 °C in a 5% CO_2_-humidified incubator. Then, the supernatant was carefully replaced with freshly prepared extracts or crude polysaccharides (0–2500 μg/ml) in complete F-12 K medium. After 48 h of incubation, 3-(4,5-dimethythiazol-2-yl)-2,5-diphenyltetrazolium bromide (MTT) assay was performed as described previously^26^. The extent of the reduction of MTT was determined by measurement of the absorbance at 540 nm with 690 nm as background absorbance with an ELISA microplate reader (Sunrise, Tecan). The complete F-12 K medium was the blank, and cells incubated in the medium only were denoted as the negative control. The 50% inhibitory concentration (IC_50_) was interpolated from the response curve.

### Assessment of neuritogenic activity of hot aqueous and ethanolic extracts, and crude polysaccharides in PC-12 cells

#### Stimulation of neuritogenesis in PC-12 cells

Cells were plated at a density of 5 × 10^3^ cells per well in 12-well plates and then treated with freshly prepared extracts or crude polysaccharides (25 to 100 μg/ml) in complete F-12 K medium. Cells treated with 50 ng/ml of NGF (Sigma) served as a positive control, while cells in complete F-12 K medium without treatment served as a negative control. Assay plates were incubated for 48 h at 37 ± 2 °C in a 5% CO_2_-humidified incubator prior to scoring the neurite-bearing cells.

#### Quantification of neurite bearing-cells

Differentiated cells were counted by visual examination of the field. A neurite-bearing cell was defined as a cell with one or more axon-like extension that was double or more the length of the cell body diameter^58^. Ten selected microscopic fields with an average of 200–300 cells per well were assessed under an inverted microscope (Nikon Eclipse TS100). The images were captured with a QImaging Go-3 color CMOS Camera (QImaging) and by the image processor system, Image-Pro Insight (MediaCybernetics). The percentage of neurite-bearing cells was evaluated by scoring the proportion of neurite-bearing cells to the total number of cells in a well.

#### Immunofluorescence staining of neurofilaments

Immunofluorescence assay was carried out according to Schimmelpfeng *et al.* (2004)^60^. Briefly, cells were seeded at a density of 5 × 10^3^ cells per well in 12-well micro-chamber (ibidi). The cells were treated with the extracts or crude polysaccharides for 48 h at 37 ± 2 °C in a 5% CO_2_-humidified incubator. After fixing with 4% paraformaldehyde, the cells were incubated with anti-neurofilament 200 antibody produced in rabbit (1:80; Sigma), and followed by fluorophore-conjugated secondary antibody, anti-rabbit IgG-fluorescein isothiocyanate (FITC) antibody produced in sheep (1:80; Sigma). Finally, the cells were mounted with ProLong® gold antifade reagent with 4-6-Diamidino-2-phenylindole (DAPI) (Life Technologies Corporation). The slides were observed under fluorescence illumination using FITC and DAPI filters and images were captured with Nikon’s Imaging Software, NIS-Elements.

### Quantification of the concentration of extracellular NGF in cell culture supernatant

Cells were plated at a density of 1 × 10^4^ cells per well in 96-well plates. The cells were treated with freshly prepared extracts or crude polysaccharides (25 to 100 μg/ml) in complete F-12 K medium for 48 h. The cell culture supernatant was collected, centrifuged at 1500 × g for 15 min and maintained at 0–4 °C prior to assay. The samples were diluted with sample diluent at a ratio of 1:2 (v/v). The amount of NGF in culture supernatant was measured by using ChemiKine^TM^ nerve growth factor sandwich enzyme-linked immunosorbent assay (ELISA) kit (Chemicon® International Inc.) according to the manufacturer’s protocol.

### Elucidation of the involvement of NGF responsive pathway in the hot aqueous extract-stimulated neuritogenesis in PC-12 cells

#### Treatment with specific inhibitors of signaling pathway

The inhibitors for Trk receptor (K252a) and MEK/ERK1/2 signaling pathway (U0126, PD98059) (all Sigma, USA) were used. Stock solutions (10 mM) of the inhibitors were prepared in dimethyl sulfoxide (DMSO) and stored at −20 °C in the dark. Final concentrations of 100 nM of K252a, 10 μM of U0126 and 40 μM of PD98059 were prepared by diluting in complete F-12 K medium before use. Cells were pre-incubated either with or without the inhibitor for one hour at 37 ± 2 °C in a 5% CO_2_-humidified incubator, respectively before the treatment with 50 ng/ml of NGF or 25 μg/ml of hot aqueous extract (the concentration that stimulated maximal percentage of neurite-bearing cells). Cells were then incubated for 48 h prior to scoring the neurite-bearing cells.

#### Quantification of protein expression of total p44/42 MAPK (ERK1/2) and phosphorylated p44/42 MAPK (Thr202/Tyr204)

Cells were pre-incubated either with or without the MEK/ERK1/2 inhibitor, U0126 for one hour at 37 ± 2 °C in a 5% CO_2_-humidified incubator, before the treatment with 50 ng/ml of NGF or 25 μg/ml of hot aqueous extract for 48 h. Cells were washed and harvested in PBS, and centrifuged at 10,000 × g for 10 min at 4 °C. Cell pellets were resuspended in cell lysis and protein extraction buffer (Thermo Fisher Scientific Inc.) with protease inhibitor cocktail and 1 mM of phenylmethylsufonyl fluoride (Sigma) on ice, and vortex every 10 min for three times. Then, the extracted proteins were centrifuged and pelleted at 10,000 × g for 10 min at 4 °C. Supernatants were aliquoted into clean micro centrifuge tubes and kept cool prior to assay. The protein expression was quantified by using the total p44/42 MAPK (ERK1/2) and phosphorylated p44/42 MAPK (Thr202/Tyr204) sandwich ELISA kits (Cell Signaling Technology) according to the manufacturer’s protocol. The absorbance was recorded at 450 nm with an ELISA microplate reader (Sunrise, Tecan). Concentration of the protein antibodies of every treatment were quantified according to the standard graph and multiplied by the appropriate dilution factor.

#### Immunofluorescence study of protein expression of phosphorylated p44/42 MAPK (Thr202/Tyr204)

Cells were pre-incubated either with or without the U0126 inhibitor for one hour at 37 ± 2 °C in a 5% CO_2_-humidified incubator before the treatment with 50 ng/ml of NGF or 25 μg/ml of hot aqueous extract. Cells were then incubated for 48 h prior to immunofluorescence staining. Protein expression of phosphorylated p44/42 MAPK (Thr202/Tyr204) (1:100; Cell Signaling Technology) detection antibody was used as the primary antibody. The slides were observed under fluorescence illumination using FITC and DAPI filters and images were captured with Nikon’s Imaging Software, NIS-Elements.

### Statistical analysis

All the experimental data were expressed as the mean ± standard deviation (SD) of triplicate values. Statistical differences between groups were assessed using one-way analysis of variance (ANOVA) of a minimum of three independent experiments and Duncan’s multiple range test (DMRT), p < 0.05 was considered to be significant.

## Additional Information

**How to cite this article**: Seow, S. L.-S. *et al.* *Lignosus rhinocerotis* (Cooke) Ryvarden mimics the neuritogenic activity of nerve growth factor via MEK/ERK1/2 signaling pathway in PC-12 cells. *Sci. Rep.* **5**, 16349; doi: 10.1038/srep16349 (2015).

## Figures and Tables

**Table 1:**
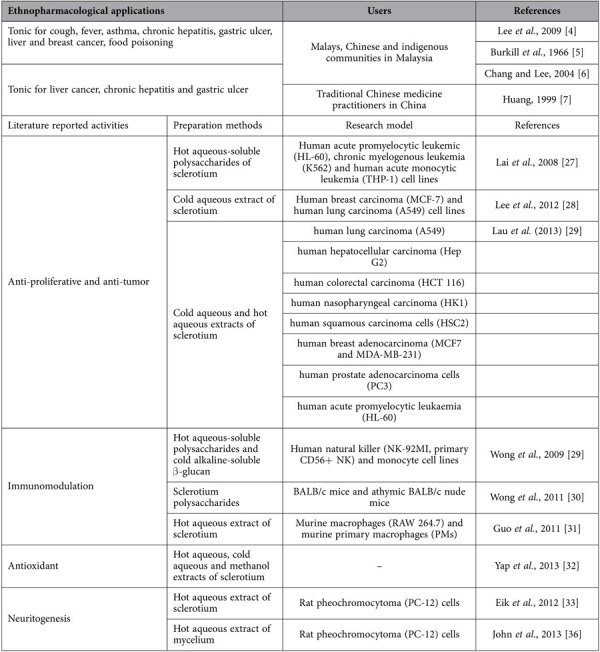
Summary of ethnopharmacological applications and literature reported activities of *Lignosus rhinocerotis*.
